# The co-existence of peripheral and vestibular neuropathy in diabetes: a cross-sectional study

**DOI:** 10.1007/s00405-023-08130-6

**Published:** 2023-07-29

**Authors:** Aksayan Arunanthy Mahalingasivam, Asger Krohn Jespersen, Niels Ejskjaer, Dan Dupont Hougaard, Peter Vestergaard, Nicklas Højgaard-Hessellund Rasmussen, Johan Røikjer

**Affiliations:** 1 Department of Clinical Medicine, Aalborg University, Aalborg, Denmark; 2 Steno Diabetes Center North Denmark, Aalborg University Hospital, Aalborg, Denmark; 3 Department of Endocrinology, Aalborg University Hospital, Aalborg, Denmark; 4 Department of Clinical Medicine, Faculty of Medicine, Aalborg University, Aalborg, Denmark; 5 Balance & Dizziness Centre, Department of Otolaryngology, Head and Neck Surgery and Audiology, Aalborg University Hospital, Aalborg, Denmark; 6 Integrative Neuroscience, Aalborg University, Søndre Skovvej 3E, 9000 Aalborg, Denmark

**Keywords:** Diabetes, Neuropathy, VOR, Vestibular

## Abstract

**Purpose:**

Diabetic neuropathy can lead to decreased peripheral sensation and motor neuron dysfunction associated with impaired postural control and risk of falling. However, the relationship between decreased peripheral sensation and impaired vestibular function in diabetes mellitus is poorly investigated. Therefore, the aim of this study was to investigate the relationship between peripheral and autonomic measurements of diabetic neuropathy and measurements of vestibular function.

**Methods:**

A total of 114 participants with type 1 diabetes (*n* = 52), type 2 diabetes (*n* = 51) and controls (*n* = 11) were included. Vestibular function was evaluated by video head impulse testing. Peripheral neuropathy was assessed by quantitative sensory testing and nerve conduction. Autonomic neuropathy using the COMPASS 31 questionnaire. Data were analyzed according to data type and distribution.

**Results:**

Measurements of vestibular function did not differ between participants with type 1 diabetes, type 2 diabetes or controls (all *p*-values above 0.05). Subgrouping of participants according to the involvement of large-, small- or autonomic nerves did not change this outcome. Correlation analyses showed a significant difference between COMPASS 31 and right lateral gain value (*ρ* = 0.23, *p* = 0.02,), while no other significant correlations were found.

**Conclusion:**

Diabetic neuropathy does not appear to impair vestibular function in diabetes, by means of the VOR.

**Clinical trials:**

NCT05389566, May 25th, 2022.

## Introduction

Diabetic peripheral neuropathy (DPN) is a common and severe complication to diabetes and the primary cause of diabetic foot ulcers with risk of subsequent amputation [1, 2]. The condition may also lead to neuropathic pain, which is associated with marked reduction in quality of life [3].

DPN leads to decreased peripheral sensation and motor neuron dysfunction, which impair the postural control system [4–8]. These changes are associated with an increased risk of falls and fractures [9].

Postural control is the ability to maintain, achieve or restore balance during any posture or activity. It is a complex chain of events, that rely on *sensory inputs* from vision, proprioception, and the vestibular system, *integration* in the brainstem and cerebellum, and *motor outputs* from the vestibular-ocular reflex (VOR), the eyes and postural adjustments [9, 10]. DPN reduces somatosensory feedback and is responsible for an inappropriate motor response, which impairs the postural control mechanisms [5, 11–13].

In fact, long-term hyperglycemia is associated with a deteriorated somatosensory system, which impairs the ability to detect postural changes to make appropriate adjustments to avoid falls [13, 14]. Long-term hyperglycemia has been proposed to cause inflammation associated with a decrease in sensitivity of the metabolic vasculature of the inner ears [13, 15]. This is also present in animal studies of long-term experimental diabetes, where significant structural damage in the otolith organ, responsible for detecting linear head acceleration, has been reported [13, 16]. However, human studies investigating the relation between diabetic neuropathy and the function of the vestibular system are limited. Some studies have shown prolonged phase lag of the VOR, while others have shown more frequent dysfunction of the vestibular system in persons with diabetes compared to persons without [13, 17, 18]. Video head impulse test (vHIT) is a diagnostic tool for assessment of vestibular function by means of VOR testing. A decrease in VOR-function indicates impairment of the vestibular system and/or vestibular nerve(s) if no other neurological signs related to structures involved in the VOR are present [19, 20]. vHIT could therefore be used as a simple diagnostic tool to assess diabetic neuropathy related to the inner ear. As the relationship between peripheral measurements of neuropathy and measurements of vestibular function in diabetes remains unexplored, our primary aim was to elucidate co-existence of peripheral and vestibular neuropathy in people with diabetes by complete (six semicircular canal) vHIT examinations.

## Methods

### Source of data

The trial was conducted at Aalborg University Hospital, Denmark, between the 4th of April 2022 and the 29^th^ of September 2022. The trial was conducted in compliance with Harmonized Tripartite Guideline for *Good Clinical Practice* (ICH GCP) [21], applicable regulatory requirements, and the Helsinki Declaration for biomedical research involving test participants [22]. The trial was reported to Clinicaltrials.gov (NCT05389566), The North Denmark Region Committee on Health Research Ethics (protocol number: N-2019-0004) and to the North Jutland Research department (ID-number of 2018-174). All data were collected and stored in a Research Electronic Data Capture (REDCap) system [23].

### Study population

The present study was a cross-sectional study consisting of adult participants with type 1 diabetes (T1D) (*n* = 52), type 2 diabetes (T2D) (*n* = 51) and controls without diabetes (*n* = 11). The participants were recruited from the DIAFALL-cohort, which is described in detail elsewhere [9]. No dropouts were registered during the study period. The small control group was included to ensure all equipment measured as expected for comparison with existing normative data.

Participants with T1D and T2D were between 40 and 80 years of age and had a diagnosis of diabetes for at least 1 year. Participants without diabetes had HbA1c-values below 48 mmol/mol (6.5%). Participants were excluded if they (1) suffered from severely decreased liver or kidney function, (2) had active cancer or previous chemotherapy, (3) were terminal ill, (4) were pregnant or breastfed, (5) did not understand written or verbal Danish, (6) had ongoing or prior alcohol- or substance abuse, (7) had extreme physical activity of more than 10 h/week, (8) had a history of vestibular impairments or (9) did actively participate in other clinical trials or (10) suffered from any neck problems.

### Large nerve fiber assessment

To assess the function of the large nerve fibers, bedside nerve conduction studies (conduction velocity and amplitude) of the sural nerve were performed on the right leg (NC-stat DPNCheck™, NeuroMetrix, Waltham, United States) [24]. If three or more attempts were inconclusive the left leg was evaluated instead.

The participant had the tested leg flexed resting comfortably on the seat of a chair, ensuring relaxed muscles. Only one successful result was recorded for each participant [24]. Biothesiometry was performed prior on the first toe according to the clinical standard. The average of both legs was presented [25].

### Small nerve fiber assessment

To assess the function of the small nerve fibers, thermal quantitative sensory testing (QST) was performed on the right leg (TSA-2001 Neurosensory Analyzer, Medoc, Ramar Yishai, Israel) [26]. A thermode was attached to the dorsal part of the right foot. The thermode increased or decreased in temperature at a controlled rate (1 °C/s) according to the protocol by the German Research Network on Neuropathic Pain [27]. The participant held a response unit to indicate when the given threshold was met. The measurements were made in the following order: Heat detection threshold, cold detection threshold (CDT), heat pain detection threshold, and cold pain detection threshold. Each variable was tested three times consecutively.

When testing, the participant sat on a chair with the right leg resting extended and elevated on a chair.

### Autonomic nerve fiber assessment

Composite autonomic symptom score 31 (COMPASS 31) was used to evaluate symptoms caused by abnormalities in the autonomic nervous system. A translated version from English to Danish was used. The questionnaire was completed on the day of the examination [28, 29].

### Video head impulse test

The VOR, and thereby the function of the vestibular system, was assessed using the ICS Impulse® (Otometrics, Denmark) vHIT system [30]. This is, to a large extent, an objective test method that evaluates the VOR function of each of the three paired semicircular canals (SCCs) within the vestibulum of the inner ear, by comparing the ratio of the eye- and head velocities (mean gain) as well as an identification of compensatory pathological saccades [10].

The test setup included the following: A hard shell, non-rotational chair positioned at a 1-m distance from the eyes being examined and a wall with a dot used for eye fixation during testing. Markings on the floor for the legs of the chair ensured consistent and reproducible positioning of the chair in zero- and 45-degree angles to the dot for horizontal- and vertical VOR testing, respectively.

An H-configuration test, prior to vHIT testing, ensured that patients did not have any eye muscle palsies and/or paralysis that could alter the test results. Prior to vHIT examination, calibration of the ICS Impulse® was performed according to the manufacturer’s guidelines. Prerequisites for each vHIT test included a minimum of 15 correctly executed head impulses for each SCC. Head impulses must ideally comply with these criteria: head impulses must be unpredictable in timing and direction, must be delivered with a peak head velocity of 150–250 degrees per second with a peak amplitude of 5–20 degrees [10].

A pathological examination was defined by the following criteria: (1) pathologically low mean gain value (below 0.80 for horizontal SCCs and below 0.70 for vertical SCCs) and (2) presence of pathological saccades in the same direction as the eye movement. Pathological saccades were defined as saccades that are present in at least 50% of all head impulses, saccades that appear in the direction as the VOR, saccades that have an amplitude of at least 50% of peak head velocity, and saccades that must appear in the interval between 100 ms (ms) after initiation of head movement to 100 ms after end of head movement (saccade window) [10].

### Statistics

Descriptive statistics were categorized as continuous data presented with a mean and standard deviation (SD) or median and interquartile range (IQR), depending on data distribution. Categorical data was reported as percentages for each group. The distribution of continuous variables was examined by histograms and Shapiro–Wilk tests of normality. For normally distributed data, a *t*-test and one-way analysis of variance (ANOVA) was performed for intergroup comparisons. The resulting *p*-value was recorded and reported together with the mean and SD for the original data.

For non-normal distributed data, a Kruskal–Wallis rank test and pairwise Mann–Whitney *U* tests with Bonferroni correction were used to assess the difference between two groups. The resulting *p*-values were recorded and reported together with the median and interquartile range for the original data.

Chi-squared tests were used on pathological vHIT measurements.

vHIT measurements and measurements of neuropathy, were correlated via Spearman rank correlation.

The mean of each QST measurement was calculated, and the difference between the calculated mean and the baseline temperature of 32 °C were then log10-transformed [31].

Cut off for the three measurement of neuropathy was set at a log10CDT > 0.687 for women and log10CDT > 0.838 for men [27], total weighted score of COMPASS 31 at 17 [11] and sural nerve conduction velocity < 40 m/s [32].

Analyses were initially performed in the three groups (T1D, T2D, and controls), and were subsequently performed in the cohorts with diabetes, sub-grouped according to neuropathy status (neuropathy and no-neuropathy) based on whether they had abnormal small fiber (cold detection), large fiber (nerve conduction) or autonomic (COMPASS 31) nerve fiber function.

Data were analyzed using the statistical software StataMP version 17.0 for Mac (StataCorp, College Station, TX). A two-sided p-value < 0.05 was considered significant.

## Results

A total of 114 participants were included and all participants completed the study (Table 1)*.* The majority of the participants completed all examinations. Nine of 570 (1.6%) examinations were not completed, primarily due to an inability to assess the sural nerve with the NC-stat DPNCheck despite several attempts. Participant characteristics are shown in Table 1.

**Table 1:** Population characteristics

Variables	T1D (*n* = 52)	T2D (*n* = 51)	Control (*n* = 11)
Age, years, (SD/IQR)	58.58 ± 1.47^§^	66.0 [57.0;72.0]	58.8 ± 3.3^§^
Sex, male, percentage	50.0	64.7	45.5
BMI, kg/m^2^, (SD/IQR)	26.1 [23.4;30.0]	30.1 ± 0.7^§^	25.2 [23.0;32.6]
HbA1c, mmol/mol, (SD/IQR) percentage	66 [59;72] 8.2%	49 [45;65] 6.6%	– –
DM duration, years, (SD/IQR)	27.8 ± 1.9^§^	11.0 [6.0;20.0]	–
Biothesiometry, volt, (SD/IQR)	15 [10;22]	24.8 ± 1.9^§^	–

Table 1 shows descriptive statistics of study population, sub-grouped in T1D, T2D and control with a count (*n*), for each variable. Due to controls not having diabetes mellitus, measurements for HbA1c, DM duration and biothesiometry are not included. Data are presented as either percentages or as mean/median values and depending on data distribution with Standard Deviation (SD) or Interquartile Range (IQR)

*T1D* Type 1 diabetes, *T2D* Type 2 diabetes, *DM* Diabetes mellitus, *BMI* Body mass index

^§^Mean (Normally distributed)

vHIT mean gain measurements did not differ significantly between T1D, T2D and controls (all *p*-values above 0.05). Total weighted score of COMPASS 31 differed significantly between T2D and controls (*p*-value < 0.01) but not between T1D and T2D (*p* = 0.55) or T1D and controls (*p* = 0.15). A significant difference was also found in sural nerve conduction velocity between T1D and controls (*p* > 0.01), and between T2D and controls (*p* = 0.02), but not between T1D and T2D (*p* = 0.42) (Table 2)*.*

**Table 2:** Neuropathy measurements

Variables	T1D (*n*)	T2D (*n*)	Controls (*n*)	*p*-value
Cold detection threshold, °C	28.0 [24.0;30.0], (51)	26.0 [21.0;28.2], (51)	28.1 [26.9;29.2], (11)	0.07
COMPASS 31, total weighted score	14.0 [3.9;28.6], (52)	18.0 [9.7;32.7], (50)	3.9 [1.3;18.1], (11)	0.02^C^
Sural nerve conduction velocity, m/s	45.0 [37.0;49,0], (51)	45.0 [38.5;52.0], (49)	50.4 ± 3.9^§^, (10)	0.01^B C^

Table 2 shows results for neuropathic measurements, sub-grouped in T1D, T2D and control with a count (n), while also presenting an intergroup p-value. Data is presented as mean/median values and depending on data distribution with Standard Deviation (SD) or Interquartile Range (IQR). p-values are performed by Kwallis

*A* significant between groups T1D/T2D, *B* significant between groups T1D/controls, *C* significant between groups T2D/controls, *T1D* type 1 diabetes, *T2D* type 2 diabetes. *p*-value = Kwallis. § = mean (Normally distributed)

Only a few participants demonstrated pathological vHIT examinations including both pathological mean gain and saccades (Table 3). No significant difference was found (*p* > 0.05) in either group.

**Table 3:** Pathological vHIT measurements

Variables	Pathological mean gain			Pathological saccades			Pathological mean gain + saccades		
	Control	T1D	T2D	Control	T1D	T2D	Control	T1D	T2D
Left lateral SCC, percentage	0.0%	2.0%	7.8%	11.1%	9.8%	7.8%	0.0%	2.0%	2.0%
Right lateral SCC, percentage	0.0%	2.0%	5.9%	11.1%	5.9%	5.9%	0.0%	0.0%	2.0%
Left anterior SCC, percentage	11.1%	9.8%	23.5%	0.0%	3.9%	0.0%	0.0%	0.0%	0.0%
Right posterior SCC, percentage	33.3%	21.6%	33.3%	11.1%	11.8%	3.9%	0.0%	3.9%	0.0%
Left posterior SCC, percentage	0.0%	2.0%	8.0%	0.0%	3.9%	0.0%	0.0%	0.0%	0.0%
Right anterior SCC, percentage	0.0%	0.0%	2.0%	0.0%	2.0%	2.0%	0.0%	0.0%	0.0%
Total, percentage	7.4%	6.2%	13.5%	5.6%	6.2%	3.3%	0.0%	1.0%	0.7%

Table 3 shows results for pathological mean gain values, saccades, and both for all six SCCs in vHIT, sub-grouped in T1D, T2D and control. Data are presented as a percentage of the total number of examinations

*T1D* type 1 diabetes, *T2D* type 2 diabetes, *SCC* semicircular canal

### Participants grouped by nerve fiber involvement

When comparing participants with diabetes (neuropathy and no-neuropathy), no significant differences were found in any parameters regardless of the type of nerve fiber involvement (small-, large- or autonomic neuropathy) or the SCCs within the vestibular system (Table 4)*.*

**Table 4:** vHIT measurements based on neuropathic status

Variables	Large fiber involvement	Small fiber involvement	Autonomic neuropathy	No large fiber involvement	No small fiber involvement	No autonomic neuropathy	*p*-value
Left lateral SCC, mean gain value	0.95 ± 0.02^§^	0.94 ± 0.01^§^	0 .96 ± 0.02^§^	0.94 ± 0.01^§^	0.94 ± 0.01^§^	0.94 [0.88;0.99]	0.8
Right lateral SCC, mean gain value	1.01 [0.96;1.07]	1.01 ± 0.02^§^	1.04 ± 0.02^§^	1.02 [0.97;1.06]	1.02 [0.99;1.06]	1.01 [0.97;1.07]	0.6
Left anterior SCC, mean gain value	0.81 ± 0.04^§^	0.83 ± 0.03^§^	0.83 [0.73;0.96]	0.87 [0.73;0.95]	0.87 [0.76;0.93]	0.85 ± 0.02^§^	0.9
Right posterior SCC, mean gain value	0.76 ± 0.03^§^	0.78 ± 0.02^§^	0.78 ± 0.02^§^	0.83 [0.70;0.88]	0.82 [0.70;0.87]	0.79 ± 0.02^§^	0.9
Left posterior SCC, mean gain value	0.94 ± 0.03^§^	0.94 [0.86;1.00]	0.92 ± 0.02^§^	0.94 [0.86;0.99]	0.95 [0.89;1.01]	0.95 [0.86;1.01]	0.9
Right anterior SCC, mean gain value	1.10 ± 0.04^§^	1.08 ± 0.02^§^	1.07 [1.00;1.17]	1.07 ± 0.02	1.06 [1.00;1.18]	1.08 ± 0.02^§^	1.0

Table 4 shows results for mean gain value for all six SCCs of participants with diabetes mellitus in vHIT sub-grouped in regard to neuropathic status, with no consideration to diabetic type. Data is presented as mean/median values and depending on data distribution with standard deviation (SD) or interquartile range (IQR). Cut off for the three measurement of neuropathy was set at a small nerve fiber involvement: log10CDT > 0.687 for women and log10CDT > 0.838 for men [27], autonomic neuropathy: Total weighted score of COMPASS 31 at 17 [11] and large nerve fiber involvement: Sural nerve conduction velocity < 40 m/s [32]

*Gain value* Eye velocity in °/s divided by head velocity in °/s, *SCC* Semicircular canal. *p*-value = kwallis

^§^ = Mean (Normally distributed)

### Correlation analyses

Total weighted score of COMPASS 31 and right lateral SCC mean gain value displayed a weak significantly correlation (ρ = 0.23, and *p* = 0.02, respectively), while the other parameters did not (all *p* > 0.05) (Table 5)*.*

**Table 5:** Correlation analyses of vHIT and neuropathy measurements

Variables	Left lateral SCC, mean gain value	Right lateral SCC, mean gain value	Left anterior SCC, mean gain value	Right posterior SCC, mean gain value	Left posterior SCC, mean gain value	Right anterior SCC, mean gain value	Sural nerve conduction velocity	Cold detection threshold, degrees Celsius	COMPASS 31, total weighted score
Left lateral SCC, mean gain value	1.0	–	–	–	–	–	–	–	–
Right lateral SCC, mean gain value	0.8*	1.0	–	–	–	–	–	–	–
Left anterior SCC, mean gain value	0.4*	0.3*	1.0	–	–	–	–	–	–
Right posterior SCC, mean gain value	0.4*	0.4*	0.8*	1.0	–	–	–	–	–
Left posterior SCC, mean gain value	0.5*	0.5*	0.3*	0.4*	1.0	–	–	–	–
Right anterior SCC, mean gain value	0.2*	0.3*	0.3*	0.2*	0.7*	1.0	–	–	–
Sural nerve conduction velocity	0.1	0.1	0.0	0.1	0.0	0.0	1.0	–	–
Cold detection threshold, degrees Celsius	− 0.1	− 0.1	− 0.1	− 0.1	− 0.1	− 0.1	− 0.2*	1.0	–
COMPASS 31, total weighted score	0.2	0.2*	− 0.0	0.0	0.0	− 0.1	− 0.1	0.1	1.0

Table 5 shows the results correlation coefficient for mean gain value for all SCCs in vHIT and neuropathic measurements. Data are presented as rho, from Spearman rank correlation analyses

^*^ significant data, *SCC* semicircular canal, Gain value: eye velocity in °/s divided by head velocity in °/s

## Discussion

This study examined the relationship between vestibular function and peripheral measurements of neuropathy in participants with T1D, T2D and healthy controls. Our study found no significant differences in vHIT between participants with T1D, T2D and controls, even after regrouping into different categories based on type of nerve fiber involvement. Despite our sub-grouping of participants with diabetes into groups with and without neuropathy, we were not able to demonstrate any association between vestibular impairment and the presence of peripheral diabetic neuropathy, thus indicating that peripheral neuropathy is not accompanied by impaired nerve function of the VOR.

### Relationship between vestibular function and peripheral nerve status

To our knowledge, this is the first study to assess the relationship between the vestibular function and extensive measurements of diabetic peripheral neuropathy in participants with T1D and T2D. Previously, a few studies with divergent results have investigated the relationship between diabetes and the vestibular system. One study compared mean gain values of all SCCs vHIT measurements between three groups: T2D without DPN (*n* = 33) with DPN (*n* = 33) and controls (*n* = 35) and found no significant difference between the groups. The study does not elaborate when a vHIT examination is pathological [19]. Another study investigated participants with T2D (*n* = 25) compared to controls (*n* = 25) and did find a significant difference in mean gain values, except the right anterior SCC. There are no mentions of saccades in the study [33]. Another study examining T1D (*n* = 15) and controls (*n* = 16) with vHIT had pathological examinations with two patients in all tested SCCs when looking at mean gain values. Saccades are not mentioned in that study. No significant difference between groups was found [34]. None of these studies investigated both T1D and T2D and the power of each study was limited. Kalkan et al. is in line with our findings regarding the relationship between neuropathic status and vestibular function [19]. In consideration of the extensive sample size of our study, it could be speculated that our results are more representative of the diabetes population, and that the negative association can truly be trusted.

During our correlation analyses between measurements of neuropathy and vHIT, only total weighted score of COMPASS 31 and right lateral SCC mean gain value showed a weak significant correlation. This in total yielded 1 of 21 correlations to be significant and must be assumed to be a random finding.

A study of T1D (*n* = 60) and healthy controls (*n* = 20), demonstrated lower gray matter volume in individuals with T1D regardless of DPN and neuropathic pain [35]. We, however, only found four participants with pathological vHIT examinations with pathological mean gain values and saccades in at least one SCC. There was no association with peripheral alterations. It is, however, interesting that no decrease was found in the T1D group despite the probability of lower gray matter volume. It could be speculated that the findings do not have any clinical significance for the brainstem function except attenuated memory in the cerebrum.

Usage of electroencephalography (EEG) might shed light on central nervous system (CNS) alterations in persons with diabetes, as it records conductivity and velocity of brain waves. As VOR includes a few structures of CNS, EEG might be more susceptible to recording dysfunction. However, to our knowledge no studies have been performed on this matter.

Future studies investigating dynamic postural control could be beneficial in understanding the increased risk of falling [9].

### Strengths

vHIT: pre-training, including vHIT related theory, and collection of data with the ICS Impulse® was done according to updated research supported guidelines and guidance of the primary author [10]. To ensure quality, consistency, and low degree of interrater variation with testing, the two primary authors of this study exclusively performed all tests. In our analysis of vHIT measurements, we interpret both mean gain values and saccades. It is the opinion of the authors of this article, that mean gain values alone is not sufficient to diagnose hypo functioning VOR with each SCC.

Large nerve fiber assessment: assessing the large nerve fiber can be done in several ways, and the golden standard is electroneurography (ENG). A study investigating the usability of DPNcheck found an excellent correlation in data output when comparing measurements from ENG and DPNcheck and a great interclass correlation. This study therefore concludes that DPNcheck has excellent reproducibility and would be a useful tool in evaluation of DPN [36]. NPC-stat was used according to the factory manual, and proper use was demonstrated by trained personnel.

Small nerve fiber assessment: QST is a method of testing the small nerve fibers for neuropathy. Studies have shown how the QST have been able to test and detect obvious neurological changes [37]. The QST was used according to the manual and pre-setting of the software provided, and we were thoroughly educated on the usage of this machinery by trained personnel. To maintain quality and consistency, the thermal plate was placed on the dorsal part of the right foot adjacent to the 2–4 metatarsal.

Autonomic nerve fiber assessment: COMPASS 31 was created as a simple tool for quantifying the severity of autonomic symptoms across several domains. Since the creation of this questionnaire, it has been validated in several studies extensively, demonstrating an association with autonomic function. The questionnaire is originally made in English. To validate the Danish translated version, it was tested on bilingual participants both healthy controls and those with autonomic alterations. It was found that the Danish translation correlated with the English version on all sub scores, and the re-test reliability found no consistent bias [28].

In our study, we use several well-known and well tested measurements for diabetic neuropathy. By using multiple measurements, we could further assess the extent of neuropathy and correlate the findings with vHIT.

A post-hoc power analysis of this study including the obtained 114 participants yielded 91.4% power. In order to reach 95% power 134 participants were needed.

### Limitations

Several limitations must be discussed. First, vHIT: VOR consists of several anatomical structures within the CNS. Therefore, to directly conclude vestibular- or vestibular nerve impairment with vHIT testing, the examiner must make sure that every other structure, involved in the VOR, must function properly. H-configuration and screening of major neurological deficits were, therefore, performed. However, because a complete neurological examination was not performed, other CNS pathologies may have been present and could thereby theoretically have altered the results.

In general, vHIT saccade measurements need interpretation by the examiner, thus increasing the probability of error and interrater variation. Mean gain values are, however, a calculated result recorded by the device and independent of the examiner. By including both the mean gain value and accompanying saccades as mandatory for the classification of hypo functioning VOR, possible weaknesses are limited such as prediction of false pathology.

Evaluating the left anterior and right posterior SCCs an increased number of pathological mean gain values occur persistently. This could be due to systematical error such as right-hand dominance of examiners. Several participants complained about lack of visual fixation of the right eye, when evaluating left anterior and right posterior SCCs, due to the bridge on the frame of the ICS Impulse®. The participants could have suffered from a latent strabismus expressed by the frame. This could have been excluded with a cover-uncover test.

QST: thus reproducibility, it is missing objectivism, and a lot of distractions influence the output [37].

Some participants were not able to detect change in temperature and only responded to pain regardless of the investigation goal. Some triggered the responder earlier or later than warranted due to both lack of attention and misunderstanding of the information given by the investigator. We did, however, record three consecutive responses per investigation goal, thus eliminating the effect of improper response on the result.

Regarding neuropathic severity, it shows T1D and T2D are more prone to have DPN than controls when looking at large nerve fiber neuropathy.

Within autonomic neuropathy, only a significant difference is seen between T2D and control. It could be speculated that the median age of T1D, being noticeably younger than T2D, influences severity.

In the measurements of small nerve fiber neuropathy, no significant difference is seen at all between the three groups.

Thus, indicating that our included participants might be less affected by the long-term consequences of diabetes and unregulated glucose level, and is therefore not as influenced by DPN in small nerve fibers and autonomic nerve fibers contradicting other studies [24, 38, 39]. When inspecting our data for vestibular impairment, no significant differences were found, even though Nourizadeh et al. concludes diabetes does affect cochlea [33]. The study, however, is limited in its power due to a lack of study participants compared to our study.

## Conclusion

Our study found no indications of a relationship between peripheral, sensory measurements of diabetic neuropathy and impaired vestibular function measured by VOR. This indicates that high frequency vestibular function is not affected by alterations in the sensory nervous system and is thus not a component in impaired balance and associated increased incidence of falls, as it is seen in diabetes. Further studies are, however, needed to confirm these findings, including more sensitive measures for impaired vestibular function.

## Data Availability

All data were collected, secured, and anonymized in the Research Electronic Data Capture (REDCap). Computer and research equipment was provided by the Balance & Dizziness Centre, Department of Otolaryngology, Head & Neck Surgery and Audiology, Aalborg University Hospital, Aalborg, Denmark and Steno Diabetes Center North Denmark, Aalborg University Hospital, Aalborg, Denmark. All equipment was password protected and stored securely.
